# Rapid and Simple Dispersive Liquid–Liquid Microextraction (DLLME) Sample Preparation for Propofol Analysis in Hair, Blood, and Urine by Gas Chromatography–Mass Spectrometry

**DOI:** 10.1002/dta.3856

**Published:** 2025-01-22

**Authors:** Sara Odoardi, Serena Mestria, Valeria Valentini, Giulia Biosa, Sabina Strano Rossi

**Affiliations:** ^1^ Department of Healthcare Surveillance and Bioethics, Forensic Toxicology Laboratory Università Cattolica del Sacro Cuore, Fondazione Policlinico Universitario A. Gemelli IRCCS Rome Italy

**Keywords:** DLLME, microextraction, post‐mortem, propofol, sample preparation

## Abstract

Propofol is a widely used anesthetic. Although considered safe, propofol‐related deaths occur, as it is sometimes abused recreationally or used to commit suicide. A simple, rapid, and reliable method for its analysis in various biological samples is needed. Sample clean‐up is a critical step in the analysis, both in terms of time and cost, indeed. Dispersive liquid–liquid microextraction (DLLME) is a simple and fast extraction based on ternary solvent mixtures that uses small volumes of solvent and sample. A DLLME extraction followed by gas chromatography–mass spectrometry (GC–MS) analysis was developed and validated for the analysis of propofol in blood, urine, and hair. The same extraction mixture of 2.5:1 methanol/chloroform was used for the different biological samples. Validation for linearity, LOD, LOQ, precision, accuracy, and recovery gave satisfactory results for the three types of biological samples included in the study, with limits of quantification of 1 μg/mL for urine, 0.2 μg/mL for blood, and 0.1 ng/mg for hair. The DLLME procedure for purification involves a small amount of solvent, thus reducing the cost and the environmental impact. In addition, a high enrichment factor is obtained, and the time for analysis is short. The method was applied to authentic post‐mortem samples for the determination of propofol in blood, urine, and hair. Also, segmental hair analysis was performed to assess chronic propofol abuse. The developed method proved to be rapid, simple, and cost‐effective for blood, urine, and hair extract clean‐up for the determination of propofol by GC/MS.

## Graphical abstract

Development and validation of an extraction method based on dispersive liquid–liquid microextraction for the analysis by GC–MS of propofol in three different biological samples (blood, urine, and hair). The proposed method is very simple and rapid. Furthermore, the method was applied to both authentic post‐mortem and living cases, showing the suitability of this extraction technique for forensic purposes.

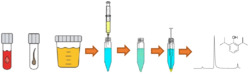

## 1 Introduction

Propofol is a drug commonly used parenterally to induce and maintain anesthesia and sedation. Propofol must be administered under the supervision of a qualified person, as patients require ventilation and monitoring during its use. Under these conditions, its use is considered safe due to its short and rapid onset of action, easy reversibility, and reduced cardiovascular and respiratory risks. Because propofol is also associated with euphoria, sexual disinhibition, relaxation, and hallucinations, it is sometimes abused recreationally [1, 2]. In particular, propofol misuse has been reported, especially among healthcare professionals such as doctors and nurses [3, 4, 5, 6, 7, 8, 9, 10, 11, 12, 13, 14]. This is because propofol is easily available in many medical settings, giving medical staff a ready access to the drug. In addition, in most countries propofol is not classified as a controlled substance. The side effects associated with propofol abuse are numerous, including hypotension, allergic reactions, vomiting, circulatory collapse, severe metabolic acidosis, cardiac arrest, and death [7, 8, 14]. Usually, propofol abuse is easier to hide than traditional drugs, so it is only discovered after the person has died, as there are no physical dependence syndromes associated with the drug use [8]. In addition, propofol is sometimes used to commit suicide [10, 11, 13].

Gas chromatography (GC) and liquid chromatography (LC) coupled to mass spectrometry (MS) are the main methods to detect propofol in biological samples. For GC–MS or GC–MS/MS, due to the high volatility of this substance, propofol can be analyzed with [9, 10, 15] or without trimethylsilyl (TMS) derivatization [11, 12, 13, 14, 15, 16], which is sometimes performed to enhance metabolite detection. In contrast, derivatization [15, 17, 18, 19, 20, 21] is required to detect propofol in LC–MS/MS with an electrospray ion source. Better ionization results can be obtained with an atmospheric pressure chemical ionization (APCI) source in negative ion mode even without derivatization [21, 22, 23].

Sample pretreatment techniques used for propofol analysis differ according to the biological sample analyzed. For blood, plasma, or urine, solid‐phase extraction (SPE) [19, 24, 25], including its micro‐version on pipette tips [18] and a dispersive SPE system such as QuEChERS [16], liquid/liquid extraction (LLE) [9, 10, 11, 15], and protein precipitation [7, 26] have been used. Hair analysis for the determination of propofol and/or its metabolite propofol glucuronide mainly involves methanolic incubation of the keratin matrix followed by LLE [14, 20], SPE [27], filtration [9, 21, 29], or evaporation and reconstitution in a more appropriate solvent [21, 28, 29].

Sample clean‐up is a crucial step in the analysis, both timewise and costwise. Dispersive liquid–liquid microextraction (DLLME) is based on ternary solvent mixtures and, unlike traditional LLE, uses small volumes of solvent and sample. In addition, this extraction is significantly faster while maintaining high efficiency. The DLLME technique has been used in several applications, including the analysis of biological specimens for the identification of analytes of interest in forensic toxicology [30, 31, 32, 33]. This study aims to develop a simple and rapid DLLME‐GC/MS method for the determination of propofol in blood, urine, and hair, its subsequent validation, and application to authentic specimens from caseworks.

## 2 Materials and Methods

### 2.1 Standards and Reagents

Water, formic acid, β‐glucuronidase/arylsulfatase from *Helix pomatia* (10.8 U/mL at 25°C for β‐glucuronidase and 25 U/mL at 25°C with 4‐NP‐sulfate for arylsulfatase), dichloromethane, sodium carbonate monohydrate, sodium bicarbonate, and methanol were purchased from Sigma‐Aldrich (Milan, Italy); ammonium formate was from Agilent (Agilent Technologies, Santa Clara, CA, USA). All reagents and solvents were of LC–MS grade.

Propofol neat standard powder was purchased from LGC (LGC GmbH, Luckenwalde, Germany). The powder was weighed and dissolved in the amount of methanol required to prepare a 1 mg/mL solution. A methanolic solution of methadone‐D3 was obtained from Chebios (Rome, Italy). Standard compounds were stored according to supplier recommendations.

A working solution for methadone‐D3 was prepared at a concentration of 0.1 mg/mL. Working solutions for propofol were prepared at 0.1 and 5 mg/mL. Working solutions were stored at −20°C until use.

### 2.2 Samples Preparation

#### 2.2.1 Hair

Hair samples were collected by cutting a strand in the posterior vertex zone with scissors, as close as possible to the scalp.

The samples were decontaminated with a single 1‐min wash with 1 mL dichloromethane with a vortex mixer. Then, the hair samples were air‐dried. A total of 50 mg of hair was cut into small pieces and extracted with 450 μL of methanol containing the internal standard (methadone‐D3, 4 μg/mg) in an ultrasonic bath for 5 h. The extract was then diluted in 2 mL of carbonate buffer (pH = 9.5).

#### 2.2.2 Blood Sample

A total of 0.5 mL of blood was added with methadone‐D3 as an internal standard (final concentration 2 μg/mL) and 0.5 mL of methanol. The sample was centrifuged at 12,000*g* for 15 min; the supernatant was collected and diluted with 1 mL of carbonate buffer (pH = 9.5).

#### 2.2.3 Urine Sample

A total of 1 mL of urine was mixed with 10 μL of concentrated formic acid and the internal standard at 1 μg/mL. The sample was hydrolyzed by incubation with 10 μL β‐glucuronidase at 40°C for 180 min. The sample was then mixed with 10 μL of 10 M sodium hydroxide solution and 0.5 mL of carbonate buffer (pH = 9.5).

#### 2.2.4 DLLME Extraction

Samples were rapidly added with a 2.5:1 methanol/chloroform mixture, 500 μL for the hair extract and 350 μL for the blood and the urine samples. After a few seconds, the samples were centrifuged for 2 min, the extraction solvents were recovered and injected into the GC–MS system.

### 2.3 GC–MS Equipment and Conditions

The GC–MS system used was an Agilent 7890 gas chromatograph coupled to an Agilent 5975C quadrupole mass detector (Agilent Technologies Italia, Milan, Italy) operating at 70 eV in electron ionization mode. The column used was a J&W 5% phenyl‐methylsilicone capillary column (30 m × 0.25 mm i.d., 0.25 μm film thickness, CPS Analitica, Mi, Italy). Helium was used as a carrier gas at a constant flow of 1 mL/min.

The temperature program was set as follows: The oven temperature was held at 80°C for 2 min, increased to 270°C at 15°C/min, and a final temperature ramp at 50°C/min to 300°C (held for 4 min). The injection port was set at 270°C in splitless mode. The mass detector was operated in SIM mode. Ions selected for propofol identification were 163, 178, and 117 (underlined is the ion used for quantification), and for its main metabolites, 149, 192, and 121 for 2,6‐diisopropyl‐1,4‐quinone and 179, 194, and 137 for 2,6‐diisopropyl‐1,4‐quinol were selected. Ion for propofol metabolites were obtained from previous publications [34, 35]. Their identification is assumed as no comparison with standards has been made.

### 2.4 Validation

The method was validated for limit of determination (LOD), limit of quantification (LOQ), linearity, precision, accuracy, and process efficiency according to SWGTOX standard practices [36].

The LOD was calculated at a concentration value that gave a signal‐to‐noise ratio S/N > 3 for three propofol characteristic fragments. This parameter was estimated based on five repeated analyses of fortified blood, urine, and hair samples at decreasing concentrations.

The LOQ was determined as the concentration in the three matrices studied that gave a signal‐to‐noise ratio equal to or greater than 10 and a CV% not exceeding 20%.

The linearity of the method was evaluated by adding the appropriate amount of propofol to the blanks extracted with DLLME to obtain six calibrators for each biological specimen, with triplicate analyses for each level. Concentration levels prepared were 1, 5, 10, 20, 50, and 100 μg/mL for urine; 0.2, 0.5, 1, 2, 3, and 4 μg/mL for blood; and 0.1, 0.2, 0.5, 1.0, 1.5, and 2.0 ng/mg for hair. Calibration curves were constructed by linear regression of the area ratio of propofol and methadone‐D3 versus nominal concentration.

Precision and accuracy were evaluated by preparing QC samples in triplicate at three different concentration levels: low (0.2 μg/mL for blood, 1 μg/mL for urine, and 0.1 ng/mg for hair), medium (1 μg/mL for blood, 10 μg/mL for urine, and 1.0 ng/mg for hair), and high (5 μg/mL for blood, 100 μg/mL for urine, and 2.0 ng/mg for hair). Precision was expressed as CV%. Accuracy was calculated as the percentage deviation from the nominal concentration normalized to the nominal concentration. The efficiency of the analytical procedure, process efficiency PE%, was calculated as the percentage of the ratio between the mean area for propofol in a QC sample prepared in triplicate and the mean area for propofol in samples prepared in the solvent of the same final volume and at the corresponding analyte concentration prepared in triplicate. This parameter was evaluated at three concentrations of the QC.

### 2.5 Authentic Samples Analysis

The validated method has been applied to authentic samples taken from post‐mortem cases in which propofol was suspected as the cause of death. Also, a hair sample from a case of a subject submitted to surgery was analyzed to evaluate the possibility of detecting a single propofol administration.

#### 2.5.1 Case 1

A 30‐year‐old female anesthesiologist was found dead at her home with a needle in her foot. The prosecutor was investigating whether the death could have been a suicide or an accident. Femoral and central (heart) blood, urine, and hair samples were taken at autopsy. The length of the hair strands was 7.5 cm. Five 1.5 cm sections were cut, numbered 1 to 5 from the proximal to the distal end, and then analyzed together with blood and urine as described above.

#### 2.5.2 Case 2

A male anesthesiologist (age 35) was found dead at his home with an IV in his arm and three empty bottles of propofol and one of midazolam next to his body. Femoral blood and urine were collected at autopsy.

#### 2.5.3 Case 3

A man (age 90) was admitted to the hospital in a critical condition. During his hospitalization, he was sedated with morphine and propofol. The patient died after 3 days. An autopsy and toxicological examination were ordered. Femoral blood was collected for toxicological analysis in the absence of a urine sample.

In all these cases, in addition to the identification and quantification of propofol, blood and urine were analyzed for ethanol, common drugs of abuse, and benzodiazepines according to routine laboratory procedures [37].

#### 2.5.4 Case 4

One of the staff personnel, submitted to sedation with propofol for a minor surgery, donated a strand of hair 1 month after the operation. The first‐centimeter hair was analyzed for propofol determination. The hair was decolorated and dyed.

## 3 Results

### 3.1 Validation Results

**FIGURE 1:**
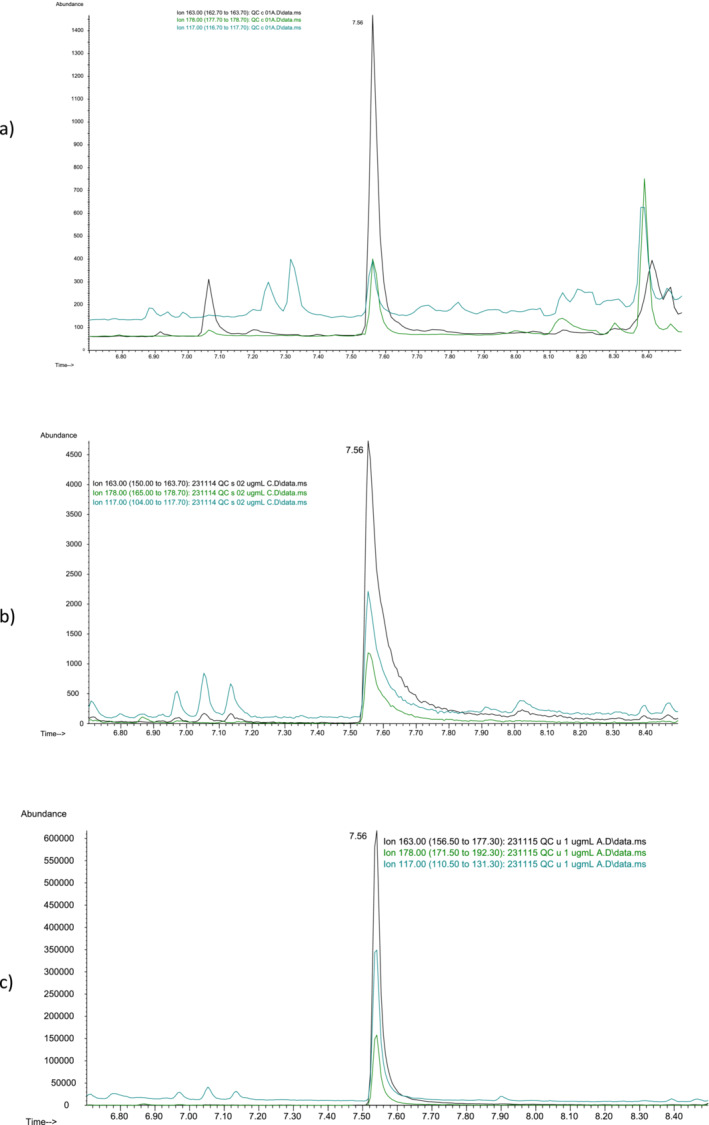
Extracted ion chromatograms of propofol in the hair (a), blood (b), and urine (c) spiked at LOQ concentrations.

The method has been validated for the three matrices of interest. LOD and LOQ were, respectively, 0.05 and 1 μg/mL for urine, 0.02 and 0.2 μg/mL for blood, and 0.05 and 0.1 ng/mg for hair. Figure 1 shows the ionic chromatograms of propofol biological samples spiked at LOQ concentrations. The method was linear in the following ranges: from LOQ to 100 μg/mL for urine, from LOQ to 5 μg/mL for blood, and from LOQ to 2.0 ng/mg for hair, with correlation coefficient *r* ^2^ always above 0.997. The CV% and the E% were all less than 18%. Process efficiency values are all satisfactory, with the results above 40%. Results for precision accuracy and process efficiency in hair, blood, and urine are shown in Table 1.

**TABLE 1:** Precision (CV%), accuracy (E%), and process efficiency (PE%) in hair, blood, and urine calculated at three concentration levels (low, medium, and high).

	CV%	E%	PE%
Hair			
0.1 ng/mg	18	10	69
1.0 ng/mg	3	14	77
2.0 ng/mg	11	2	92
Blood			
0.2 μg/mg	8	−15	47
2.0 μg/mg	5	1	41
4.0 μg/mg	11	5	43
Urine			
1.0 μg/mg	5	9	59
10 μg/mg	7	3	48
100 μg/mg	7	7	64

### 3.2 Authentic Case Results

#### 3.2.1 Case 1

Blood and urine were negative for ethanol, drugs of abuse, and benzodiazepines. Propofol was detected in all the biological samples analyzed, including all the hair sections. Concentrations were 1.7 μg/mL in femoral blood, 3.9 in heart cavity blood, 55 μg/mL urine, and 0.8–2.0 ng/mg hair (1.2; 0.8; 1.3; 2; 0.85 ng/mg from proximal to distal section). Propofol metabolites 2,6‐diisopropyl‐1–4‐quinol and 2,6‐diisopropyl‐1–4‐quinone were also detected in urine. There was no ascending or descending trend in the concentrations detected in the hair samples.

**FIGURE 2:**
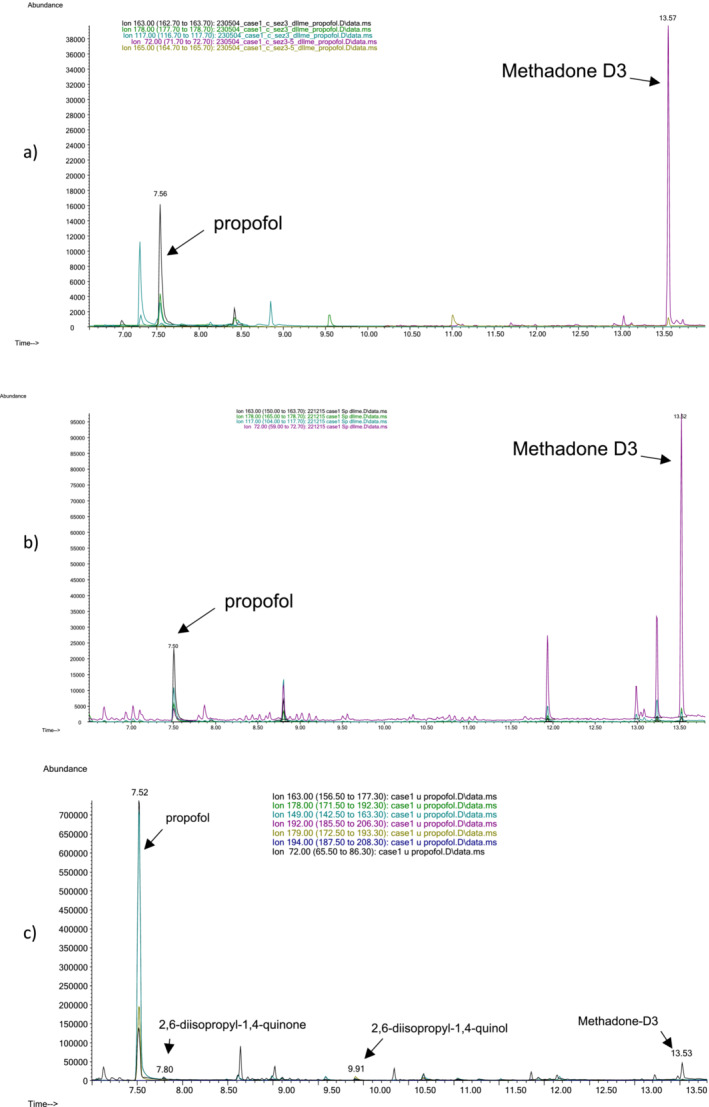
Extracted ion chromatograms of propofol in the hair (a), blood (b), and urine of Case 1.

Figure 2 shows the ionic chromatograms of propofol in the hair (a), blood (b), and urine (c) of Case 1.

#### 3.2.2 Case 2

Propofol was at 40 μg/mL in blood and at 16 μg/mL in urine. Midazolam was at 29 ng/mL, together with traces of its metabolite hydroxy‐midazolam. No ethanol or other psychotropic substances were detected in blood and urine.

#### 3.2.3 Case 3

In Case 3, propofol was found in the blood of the subject at a concentration of 0.7 μg/mL, and morphine at 13 ng/mL. No other substances were found among the drugs tested.

#### 3.2.4 Case 4

The first‐centimeter hair, analyzed for propofol determination, showed traces of propofol below LOQ, while no metabolites were identified. The low amount of propofol detected could also be due to the decoloration of the hair.

A summary of the results for authentic cases is provided in Table 2.

**TABLE 2:** Summary of authentic cases analyzed.

	Sex	Age	Propofol			Additional findings
			Blood (μg/mL)	Urine (μg/mL)	Hair (ng/mg)	
Case 1	F	30	1.7 (femoral b.) 3.9 (heart cavity b.)	55	1.2; 0.8; 1.3; 2; 0.85	
Case 2	M	35	40	16	/	Midazolam
Case 3	M	90	0.7	/	/	Morphine
Case 4	F		/	/	< LOQ	

## 4 Discussion

Sample preparation is a critical step in the analysis of biological specimens.

It is a significant source of error and affects the time and cost of the analysis. Therefore, the technique used to prepare the sample should be as fast, simple, and effective as possible.

DLLME extraction meets these requirements because it is easy, fast, and uses small volumes of extraction solvents. This method allows multiple samples to be prepared simultaneously in a few minutes. With respect to other techniques used for the pretreatment of biological samples for the detection of propofol, DLLME did not require any device to be purchased, unlike SPE, and the amount of solvent used is lower than that of traditional LLE, reported to be around 4–5 mL [9, 10, 15, 20]. This reduced amount of extraction solvent did not need to be evaporated, further reducing the analysis time. In addition, the same extraction procedure was used in the present study for the purification of the three different matrices, blood, urine, and hair extract, except for the volume of the extraction mixture used. The sensitivity of the method for the three biological samples included in the study was comparable to some of those reported in other publications [7, 13, 15], whereas in some cases, especially for hair analysis, the proposed method showed a higher LOD [18, 20, 27, 28]. The lower sensitivity of the proposed method does not seem to be related to the sample preparation technique used but to the analytical technique chosen for detection, LC–MS/MS versus GC–MS. In fact, the GC–MS methods had similar LODs to those reported in this study. Despite the higher LOD for propofol, the DLLME method with GC–MS detection was still able to detect propofol in the authentic cases analyzed, even in the case of a single administration, as in the hair of Case 4, where the amount of analyte was still detectable but below the LOQ. Consequently, the sensitivity achieved was satisfactory, resulting in LOD and LOQ being adequate for propofol determination for forensic purposes. In addition, the use of GC–MS equipment, which is affordable for the majority of laboratories, makes the method economically attractive.

### 4.1 Authentic Case

In Cases 1 and 2, the drug was self‐administered, whereas in Case 3, hospital staff administered it.

In Case 1, the blood level of propofol was within the therapeutic range [38], being 1.7 μg/mL. However, the reported therapeutic range for propofol is based on monitored, ventilated patients. The same concentration may be fatal in unventilated subjects, as reported in the literature [5]. Mean propofol hair concentration in this case was similar to those measured by other authors. [6, 12, 21, 28]. This may demonstrate the usefulness of the DLLME method for the pretreatment of hair samples for propofol determination. Propofol presence in the entire length of the hair, with a fairly constant concentration in each 1.5 segment, is consistent with long‐term use of this substance, rather than with a suicidal intent. It is therefore possible to hypothesize that the death was accidental in a habitual user of propofol.

Case 2 was, instead, a clearly suicidal behavior, with many doses of propofol and with midazolam self‐administration. The concentrations of propofol found in blood and urine were in the toxic ranges, being 40 μg/mL in blood and 16 μg/mL in urine, together with midazolam.

In Case 3, propofol blood concentration was below the therapeutic range (0.7 μg/mL).

The application of DLLME coupled to GC/MS to authentic cases, after method validation, demonstrated itself as a reliable, rapid, and quite inexpensive technique for the determination in different bodily fluids (blood and urine) and in the hair matrix.

In particular, segmental hair analysis allows one to discriminate chronic drug use with respect to therapeutic exposure.

The identification of propofol in a hair sample from Case 4 demonstrates the ability of the method to determine a single therapeutic propofol administration, as in the case of a surgical intervention.

## 5 Conclusions

This study has provided a rapid and straightforward extraction method for the determination of propofol in authentic biological specimens. DLLME proved to be simple and easy to apply to three different and complex biological samples, such as blood, urine, and hair extract. The method has therefore been applied to different authentic post‐mortem cases and to a case of a single exposure to propofol in a surgical setting, with satisfactory results.

## Conflicts of Interest

The authors declare no conflicts of interest.

## Data Availability

The data that support the findings of this study are available on request from the corresponding author. The data are not publicly available due to privacy or ethical restrictions.
